# Dual-Specificity Protein Phosphatases Targeting Extracellular Signal-Regulated Kinases: Friends or Foes in the Biology of Cancer?

**DOI:** 10.3390/ijms26178342

**Published:** 2025-08-28

**Authors:** Alessandro Tubita, Dimitri Papini, Ignazia Tusa, Elisabetta Rovida

**Affiliations:** Department of Experimental and Clinical Biomedical Sciences “Mario Serio”, University of Florence, 50134 Florence, Italy; alessandro.tubita@unifi.it (A.T.); dimitri.papini@unifi.it (D.P.); ignazia.tusa@unifi.it (I.T.)

**Keywords:** dual-specificity protein phosphatase, mitogen-activated protein kinase, cancer, targeted therapy, ERK1/2/5

## Abstract

Dual-specificity protein phosphatases (DUSPs) are a family of proteins that dephosphorylate both phospho-serine/threonine and phospho-tyrosine residues of Mitogen-Activated Protein Kinases (MAPKs). MAPKs are involved in a large number of cellular processes, including proliferation, differentiation, apoptosis, and stress responses. Therefore, dysregulation or improper functioning of the MAPK signalling is involved in the onset and progression of several diseases, including cancer. Likewise, dysregulation of DUSPs markedly affects cancer biology. The importance of MAPKs in the modulation of tumour development has been known for a long time, and MAPKs are consistently used as molecular targets for cancer therapy. However, in the last decade, DUSPs have acquired a greater interest as possible therapeutic targets to regulate MAPK activity and to prevent resistance mechanisms to MAPK-targeting therapies. Moreover, the possibility of exploiting DUSPs as biomarkers for the diagnosis and prognosis of specific types of cancer is also emerging. In this review, we report what is known in the literature on the role of DUSPs in cancer onset and progression, focusing on those targeting the extracellular signal-regulated kinases (ERKs), in particular ERK1/2 and ERK5 conventional MAPKs. The specific role of each ERK-targeting DUSP in supporting or hampering cancer progression in the context of different types of cancer is also discussed.

## 1. Introduction

Mitogen-activated protein kinases (MAPKs) are involved in a number of physiological processes and are activated by a large variety of stimuli. There are four conventional MAPKs, including the extracellular signal-regulated kinase 1 and 2 (ERK1/2), c-Jun N-terminal kinase (JNK) 1-3, p38 (α, β, γ, and δ), and ERK5. Atypical MAPKs are ERK3, ERK4, ERK8 (also known as ERK7), and the Nemo-like kinase. MAPKs phosphorylate specific serines and threonines of target proteins to turn on or off the activity of the substrate protein and regulate cellular activities ranging from gene expression, survival, and proliferation to migration, metabolism, and apoptosis. Because of the many important cellular functions controlled by MAPKs, they have been studied extensively to define their roles in cellular physiology and human diseases [1,2].

Dual-specificity protein phosphatases (DUSPs, also referred to as mitogen-activated protein kinase phosphatases (MKPs) are a subfamily of protein tyrosine phosphatases (PTPs) implicated in the regulation of MAPKs in mammalian cells. They act by dephosphorylating both tyrosine and threonine residues at the T-X-Y motif located within the activation loop of the substrate kinases [3]. Through their catalytic activity, DUSPs contribute to the fine tuning of MAPK signalling, which is achieved by ensuring appropriate amplitude and duration of their activation, and play critical physiological roles by participating in the control of key biological processes, including cell proliferation, differentiation, immune responses, adaptation to stress, and development [4,5]. 

Structure-wise, all DUSPs perform their function thanks to the presence of a conserved phosphatase domain containing aspartic acid, cysteine, and arginine residues in the catalytic site. A subset of DUSPs (namely DUSP1, DUSP2, DUSP4, DUSP5, DUSP6, DUSP7, DUSP8, DUSP9, DUSP10, DUSP16, and DUSP24) contains an N-terminal region composed by two CDC25 homology 2 domains. Additionally, based on the presence or absence of the kinase-interacting motif (KIM), a cluster of basic amino acids that mediates enzyme–substrate interaction by binding to the common domain (CD) of MAPKs, DUSPs are generally divided into two groups. The KIM-containing DUSPs, also referred to as “typical” DUSPs, include DUSP1, DUSP2, DUSP4, DUSP5, DUSP6, DUSP7, DUSP8, DUSP9, DUSP10, and DUSP16, while those (DUSP3, DUSP11, DUSP12, DUSP13, DUSP14, DUSP15, DUSP18, DUSP19, DUSP21, DUSP22, DUSP23, DUSP24, DUSP26, DUSP27, and DUSP28) that do not contain the KIM domain are considered atypical DUSPs [6,7]. DUSPs belonging to the typical group can be further divided into three subgroups based on sequence homology, intracellular localization, and substrate specificity. The first group includes DUSP1, DUSP2, DUSP4, and DUSP5, which are inducible nuclear proteins, while the second is formed by the cytoplasmic ERK-selective DUSP6, DUSP7, and DUSP9. The last group includes DUSP8, DUSP10, and DUSP16, which preferentially inactivate JNK and p38 MAPKs, and are located in both the cytoplasm and the nucleus [8].

MAPKs are often deregulated in various types of cancer. Since DUSPs are closely involved in their regulation, several studies have been carried out to shed light on the possible role of the latter in tumour development. Importantly, DUSP expression is often deregulated by multiple pathophysiological factors, including MAPKs themselves, that may be altered along cancer onset and progression. Among others, hypoxia can reduce DUSP expression via hypoxia-inducible factors [9,10], while oxidative stress modulates DUSP levels through redox-sensitive pathways, impacting DNA damage responses and apoptosis [11]. Furthermore, oncogenic signals such as Ras, Src, and NF-κB [12,13], as well as inflammatory stimuli like cytokines and growth factors [14,15] regulate DUSP expression, shaping MAPK activity and contributing to tumourigenesis. These findings highlight the complex interplay between DUSPs and signalling pathways relevant for cancer biology.

**Figure 1:**
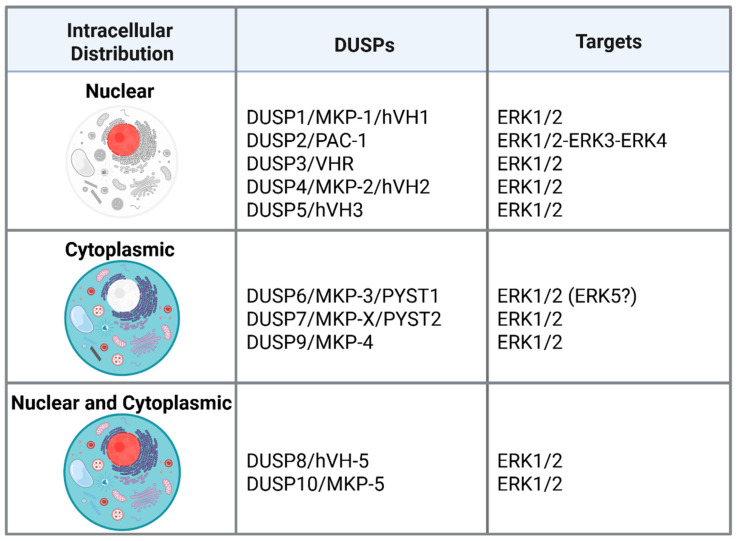
Schematic representation of ERK-targeting DUSPs involved in cancer biology. https://biorender.com/sx2f5p8 (accessed on 25 July 2025).

Here, we review the contribution of DUSPs in MAPK regulation in cancer, focusing on ERK-targeting DUSPs (Figure 1). Noteworthy, this represents only in part the contribution of DUSPs to cancer biology, considering the pivotal role of these phosphatases on p38 and JNK MAPKs. However, the aim of this review is to offer a new perspective in the field by proposing the modulation of DUSPs as an additional strategy to target canonical ERKs.

## 2. Role of ERK-Targeting DUSPs in Cancer

### 2.1. DUSP1

DUSP1, also referred to as MKP-1, was the first DUSP to be discovered and participates in the inactivation of several MAPKs, including JNK, ERK1/2, and p38 [16,17,18,19]. DUSP1 is broadly expressed in various tissues, and its expression is regulated by a wide variety of stimuli, such as cellular stress, cytokines, lipopolysaccharide, and glucocorticoids [20,21,22]. Together with DUSP2, DUSP4, and DUSP5, DUSP1 belongs to the family of inducible nuclear DUSPs.

Several lines of evidence seem to indicate that DUSP1 may support cancer onset and development. An early report on breast cancer (BC) showed that DUSP1 expression is associated with poorly differentiated or late-stage tumours [23]. Consistently, subsequent studies showed that DUSP1 is expressed at low levels in normal breast tissue and ductal hyperplasia, while it is increased in carcinoma in situ and is overexpressed in approximately 50% of infiltrating BC tumours [24]. Among the mechanisms that may support the increased expression of DUSP1 in BC, KLF5, the high expression of which has been associated with shorter survival in BC patients [25], is a possible candidate. Indeed, KLF5 overexpression increased DUSP1 protein levels in oestrogen receptor (ER)-positive MCF7 and triple-negative (i.e., lacking ER, progesterone receptor, and HER2 overexpression) BC (TNBC) Hs578T cells. Consistently, KLF5 ablation determined the downregulation of DUSP1 as well as the increase of apoptosis in immortalized mammary epithelial MCF10A cells and the TNBC BT20 cell line. Mechanistically, DUSP1 protein levels were regulated by KLF5 via MEK1/2–ERK1/2-dependent phosphorylation and proved to be necessary and sufficient for KLF5-supported cell survival [26]. The upregulation of DUSP1 by tumour-supporting genes has also been described in a study performed in TNBC MDA-MB-231 and MDA-MB-468 cells, showing that β2-adrenergic receptor, which may act as a protumoural gene [27], determines an increase in DUSP1 and a reduction of ERK1/2 phosphorylation. The latter was DUSP1 dependent since it was prevented by either genetic or pharmacological (with the DUSP1/6 inhibitor BCI [28]) DUSP1 inhibition [29]. Finally, using a BCI-derivative, it was later demonstrated that BCI-215 induces apoptosis in MDA-MB-231 cells and sensitizes them to immune cell-mediated killing. In these cells, treatment with BCI-215 activated ERK1/2 signalling without inducing reactive oxygen species (ROS)-mediated DNA damage, suggesting promising potential for clinical translation. Consistently, BCI-215 did not exhibit toxicity in zebrafish embryos or epithelial cells [30].

As far as colon cancer (CC) is concerned, although an early study reported that DUSP1 is overexpressed in the initial stages of carcinogenesis and gradually downregulated during tumour progression [23], several lines of evidence point to a protumoural role of DUSP1 in CC. In a study performed using HCT116 cells, glucose deprivation resulted in ERK1/2-dependent apoptosis. However, AMPK-dependent increased levels of DUSP1/2 protected HCT116 cells from glucose deprivation by suppressing the pro-apoptotic effect of ERK1/2 [31]. In another study, Lee and colleagues focused on a possible relationship between the topoisomerase I inhibitor camptothecin (CPT; [32]) and DUSP1 and ERK1/2 phosphorylation in CC cells. Treatment with CPT induced apoptosis in HCT116 cells and determined a marked decrease in DUSP1 expression with a concurrent increase in ERK1/2 phosphorylation and nuclear localization. Suppression of ERK1/2 activity with MEK inhibitors (U0126 and PD98059; [33]) decreases CPT-induced apoptosis. Taken together, these findings seem to indicate that DUSP1 inhibition, and the related ERK1/2 activation, could be of potential interest for the treatment of CC [34]. Accordingly, NSC 95397, a quinone-based inhibitor of DUSP1 and DUSP6 [35], reduces the viability and induces apoptosis of CC cells (SW480, SW620 and DLD-1) by increasing p21 expression and caspase-3 activity via enhanced expression of ERK1/2 [36]. Using a model of BALB/c-foxn1nu nude mice and two different cell lines (namely HCT116 and SW480), it was shown that DUSP1 promotes colorectal cancer (CRC) growth in vivo (Table 1). 

Overexpression of DUSP1 determined the inactivation of the ERK1/2 and p38 signalling pathways. Conversely, the long non-coding RNA CDKN2B-AS1, shown to target DUSP1 in vitro and in vivo, leads to the suppression of CRC growth by activating the ERK1/2 pathway [37]. Finally, as for malignant peripheral nerve sheath tumours (MPNSTs), knock down (KD) of DUSP1 and/or DUSP6, which are highly expressed in these tumours as compared to normal tissue, leads to reduced MPNST cell growth and to increased ERK1/2 phosphorylation. In vivo, administration of the DUSP1/DUSP6 inhibitor BCI increased ERK1/2 and JNK activation, caused tumour necrosis and fibrosis, and reduced tumour volume in MPNST xenografts established with S462.TY cells or patient-derived cells [38].

Concerning a possible role of DUSP1 in the support of cancer aggressiveness, it has also been reported that this DUSP supports drug resistance. Indeed, the overexpression of DUSP1 rendered non-small cell lung carcinoma (NSCLC) H460 cells resistant to cisplatin, while its downregulation sensitized these cells to cisplatin-induced cell death. Interestingly, in both NSCLC H460 and ovarian cancer (OC) OVCAR3 cell lines, cisplatin induced DUSP1 expression through a post-transcriptional mechanism regulated by ERK1/2. Another study conducted in NSCLC cell lines (PC-9 and HCC827) demonstrated that the overexpression of DUSP1 is responsible for the resistance to the EGFR tyrosine kinase inhibitor osimertinib by inhibiting ERK1/2 phosphorylation. DUSP1 KD reverted this effect and restored the sensitivity to osimertinib [65]. In line with the protective role of DUSP1 in cisplatin-induced toxicity, apoptosis induced by the latter was marked in DUSP1(−/−), while being minimal in DUSP1(+/+) mouse embryonic fibroblasts. Mechanistically, the induction of DUSP1 by cisplatin correlated with inactivation of JNK but not of ERK1/2 or p38 [66]. To clarify the role of DUSP1 in cisplatin-resistance, in a subsequent work, the same authors showed that in OC cell lines (OVCA432, TOV112D, CAOV3, OVCA420, OV433, and RMG-1) cisplatin induces DUSP1 through ERK2-mediated phosphorylation. Indeed, impairment of ERK2 activation (by the MEK1/2 inhibitor U0126) or expression (by siRNA) prevented cisplatin-induced DUSP1 expression and enhanced cisplatin-induced cell death. The latter effect was also achieved upon KD of either ERK2 or DUSP1, likely by decreasing the level of Bcl-2 protein. Collectively, these results suggested that targeting ERK1/2-DUSP1 signalling could overcome cisplatin resistance in OC [67]. Finally, in another study, the same authors demonstrated that DUSP1 KD by shRNA in CAOV3 cells increased both basal and rapamycin-induced autophagic flux, while DUSP1 overexpression had the opposite effect. Interestingly, cisplatin-resistant CAOV3 (CAOV3-CR) cells exhibited elevated DUSP1 expression levels and were refractory to rapamycin-induced autophagy and cytostatic effects. DUSP1 KD in CAOV3-CR cells restored sensitivity to rapamycin. Although it had already been shown that DUSP1 supports autophagy, this work suggests that suppression of DUSP1 may enhance the therapeutic activity of rapamycin [68]. In addition, providing evidence of the role of DUSP1 in supporting cisplatin resistance, the above studies indicate that DUSP1 supports the malignant phenotype of OC cells. A more recent study supports this role of DUSP1 in OC. Indeed, chondroitin sulfate N-acetylgalactosaminyltransferase-2 (CSGALNACT2), a Golgi transferase, downregulated the levels of DUSP1 and the phosphorylation of ERK1/2 while inhibiting cell migration and invasiveness [69]. DUSP1 has also been associated with AKT-targeting agents. In a study performed using cell lines from urothelial bladder cancer (BLCA), a tumour in which 50–70% of cases show activation of the PI3K/AKT/mTOR signalling [70], the pan-AKT inhibitor MK-2206 decreased cell viability in a number of cell lines. However, MK-2206-sensitive and -resistant cell lines showed opposite responses as for the levels of ERK1/2 phosphorylation, with a reduction of the latter in the sensitive ones and an increase in those that were resistant. The possibility that this effect was due to DUSP1 was supported by the occurrence of an inverse relationship between DUSP1 expression and ERK1/2 phosphorylation in all the analysed cell lines and by the fact that KD of DUSP1 reverted the effect of MK-2206 [39].

Early evidence supporting a possible tumour suppressor role of DUSP1 demonstrated that its expression, together with the subsequent ERK1/2 dephosphorylation, promotes apoptosis in BC. Indeed, exposure to nitric oxide (NO) determined an increase in DUSP1 transcripts and a decrease in ERK1/2 phosphorylation, and induced apoptosis in ER-positive ZR7530 cells and in TNBC MDA-MB-468 cells. On the contrary, NO failed to increase DUSP1 expression, ERK1/2 inactivation, and apoptosis in TNBC MDA-MB-231 cells [71].

Some studies provided evidence of the role of DUSP1 as an oncosuppressor in NSCLC. In an early paper, the authors assessed DUSP1 expression and ERK1/2 phosphorylation using immunohistochemistry and RT-PCR analysis, respectively, in samples obtained from twenty patients with lung squamous cell carcinoma (SCC) and five normal lungs. Expression of DUSP1 negatively correlated with tumour differentiation, and, accordingly, both the phosphorylation and expression of ERK1/2 positively correlated with tumour differentiation. This study suggested that DUSP1 may be a candidate as a marker for the prognosis of lung SCC [72]. In further support of the negative role of DUSP1 in supporting lung cancer cell proliferation, treatment of mice with a γ-secretase inhibitor (LY-411575) reduced KRas(G12V)-driven NSCLC growth, while upregulating DUSP1 protein level and decreasing ERK1/2 phosphorylation. Carcinomas from treated mice presented reduced levels of the transcriptional repressor HES1, which was shown in vitro to directly bind to and repress the DUSP1 promoter [73]. DUSP1 expression has also been associated with gefitinib sensitivity. Indeed, in NSCLC PC9 cells, DUSP1 overexpression abrogated long non-coding RNA CASC9-induced resistance to gefitinib and reduced ERK1/2 phosphorylation [74].

Concerning hepatocellular carcinoma (HCC), accumulating evidence indicates that DUSP1 may counteract tumourigenesis, making it a possible target in this neoplasm. In a first report, Liu and colleagues showed that hypoxia enhances DUSP1 transcription in HepG2 cells. DUSP1 KD enhanced HIF-1α phosphorylation and transcriptional activity, as witnessed by the increased expression of the HIF-regulated gene erythropoietin [75]. In a later work, the same authors identified the underlying mechanisms, showing that suppression of DUSP1 facilitates the interaction between the HIF-1α subunit and the coactivator p300, likely by increasing ERK1/2 phosphorylation. Indeed, treatment with the MEK1/2 inhibitor PD98059 prevented the increase in HIF-1 activity caused by DUSP1 suppression. Taken together, the above results suggest that hypoxia-induced DUSP1 protects overactivation of HIF-1 through the inhibition of ERK1/2 kinase activity [76]. In line with an antitumoural role of DUSP1, HCC patients with poorer prognosis showed low DUSP1 protein levels. DUSP1 inactivation was due to either ERK1/2–SKP2–CKS1-dependent ubiquitination and subsequent DUSP1 degradation or promoter hypermethylation associated with loss of heterozygosity at the DUSP1 locus. Functional studies revealed that DUSP1 reactivation led to the suppression of ERK1/2–SKP2–CKS1 activity, inhibition of proliferation, and induction of apoptosis in human hepatoma cell lines 7703, HuH7, SNU-182, and SNU-387 [77]. Interestingly, in a transgenic rat model expressing a dominant-negative mutant of Cx32 (major hepatocyte gap junction protein) in the hepatocytes, the downregulation of DUSP1 with consequent upregulation of ERK1/2 led to an increase of ethanol-related hepatocarcinogenesis. In particular, oral ethanol administration promoted hepatocarcinogenesis with a higher incidence in transgenic compared with WT rats, pointing to an important antitumoural role of DUSP1 in vivo [40]. Another study performed using HepG2 and HBV-genome-transfected HepG2 (HepG2.2.15) cells suggested that hepatitis B virus X (HBx) protein may support HCC development by inhibiting DUSP1 [78]. Thus, in HCC, DUSP1 may represent a valuable prognostic marker as well as a potential therapeutic target.

Additional lines of evidence have been collected in support of an antitumoural role of DUSP1 in other types of cancer. With regard to endometrioid adenocarcinoma (EEA), a type of endometrial cancer (EC), IHC analysis on tumour and normal tissue provided evidence that DUSP1 downregulation correlates with advanced stages. Accordingly, it was found that the Ishikawa cell line was the only one with high levels of DUSP1 mRNA and protein among a number of EEA cell lines (Hec1A, Hec1B, RL952, and Ishikawa). In the former, DUSP1 silencing promoted cell proliferation and migration along with an increase in ERK1/2 phosphorylation [79]. DUSP1 is also crucial in prostate cancer (PC) since its expression is downregulated in advanced and metastatic carcinomas [80]. This is consistent with another study in which it was shown that most of the apparently normal glands, benign prostatic hyperplasia, and low-grade prostatic intraepithelial neoplasia (PIN) samples show high DUSP1 expression, while DUSP1 expression levels are low or even absent in high-grade PIN and PC IHC samples [81]. These data are at variance with an earlier report performed using in situ hybridization in 50 cases, showing that DUSP1 mRNA is overexpressed in high-grade prostatic intraepithelial neoplasia (PIN) compared with normal tissue [82]. A correlation between DUSP1 and the expression of SNAIL, ERK1/2, JNK, and p38 in cell lines (DU145 and PC3) and tissue samples from patients diagnosed with benign prostatic hypertrophy or PC has also been reported. DUSP1 KO caused an increase in SNAIL and ERK1/2 phosphorylation and led to increased tumour growth and invasion, while DUSP1 overexpression decreased tumour growth [83]. More recently, in line with a possible antitumoural activity of DUSP1, Doronzo and colleagues found that in D4M BRAFV600E murine melanoma cells, TFEB silencing prevents DUSP-1 inactivation and reduces ERK1/2 activity and tumour growth, highlighting its critical role in melanoma cell proliferation and metabolic adaptation [84]. Concerning the role of DUSP1 in oesophageal squamous cell carcinoma (ESCC), it was demonstrated that overexpression of the circadian transcription factor ARNTL (also known as BMAL1) upregulates the expression of DUSP1 by inducing its transcription. This overexpression of DUSP1 blocks the phosphorylation of ERK1/2 and mediates anti-cancer activity in both in vitro and in vivo models [41]. Finally, Cheng and colleagues found that the administration of baculovirus-derived recombinant DUSP1 reduced the level of ERK1/2 phosphorylation in cell lines from different types of cancer (GC-7901, MCF-7, HeLa, A49, PC-2, and HepG2) and decreased the proliferation of HeLa cells [85].

**Figure 2:**
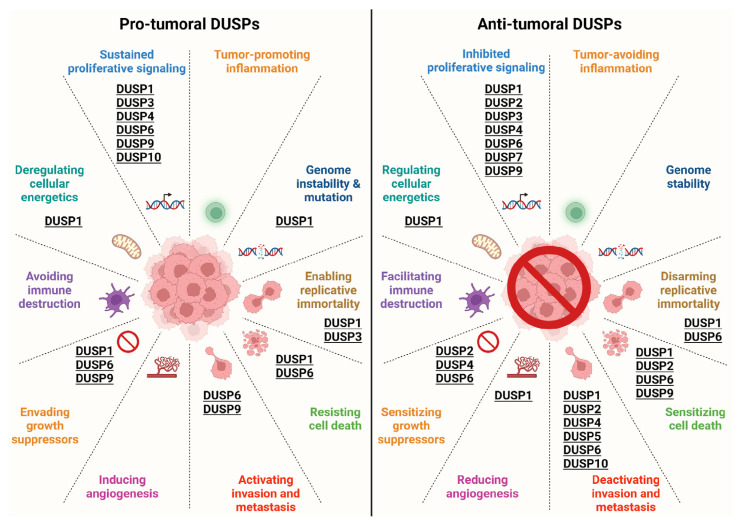
Role of DUSPs in the hallmarks of cancer. https://biorender.com/n0m03f4 (accessed 25 August 2025).

DUSP1 is one of the first discovered and most studied DUSPs, and its role in cancer has been extensively investigated. However, the mechanisms responsible for its impact on cancer onset and progression have not been fully elucidated. Furthermore, DUSP1 may support both protumoural and antitumoural activities depending on the type of cancer, as it has, for example, a clear antitumoural role in NSCLC and HCC and a protumoural role in OC and BC. However, its function in melanoma and endometrial cancer needs further clarification to understand its possible therapeutic value. The modulation of DUSP1 activity may thus be included among the possible therapeutic strategies in a subset of cancers but not in others (Figure 2).

### 2.2. DUSP2

DUSP2 is a nuclear protein widely expressed in various tissues, and its expression is regulated by numerous stimuli, including cellular stress, cytokines, lipopolysaccharide, and glucocorticoids. DUSP2 participates in the inactivation of MAPK signalling components, including ERK1/2, p38, ERK3, and ERK4 [86,87,88,89].

Lin and colleagues were the first to provide evidence for an antitumour role of DUSP2, demonstrating that its expression is markedly diminished or absent in many human cancers, with expression levels inversely correlating with both HIF-1α expression and tumour malignancy. Experiments conducted on cancer cell lines (HeLa, HCT116, and Hep3B) indicated that HIF-1α inhibits transcription and translation of DUSP2, leading to sustained ERK1/2 phosphorylation and increased chemoresistance. Consistently, DUSP2 KD enhanced drug resistance under normoxia, while the forced expression of DUSP2 abolished hypoxia-induced chemoresistance to paclitaxel, cisplatin, and oxaliplatin in vitro and in vivo. These results demonstrate that DUSP2 is a key downstream regulator of HIF-1-mediated tumour progression and chemoresistance. Thus, among the possible beneficial outcomes of HIF-targeting, we may add the re-expression of DUSP2 [9]. Along this line, a later study confirmed that HIF-1α negatively regulates the expression of DUSP2 in BC cells, while increasing ERK1/2 phosphorylation. Genetic inhibition of DUSP2 led to lapatinib resistance, while its overexpression increased cell sensitivity to lapatinib. Interestingly, low expression of DUSP2 and high expression of HIF-1α were associated with poor prognosis in ER-negative BC patients, further supporting the relevance of a HIF1α/DUSP2/ERK axis in cancer [90]. An antitumoural role of DUSP2 has also been reported in pancreatic ductal adenocarcinoma (PDAC). Indeed, overexpression of Mir-361-3p in PDAC cells led to increased EMT and invasion, but not proliferation, along with downregulation of DUSP2 and increased ERK1/2 phosphorylation. DUSP2 overexpression led to inactivation of the ERK1/2 pathway and to a reduction of Mir-361-3p-induced EMT [42]. A recent paper reporting IHC analysis of 164 gastric cancer (GC) cases revealed that high expression of SKA3 (spindle and kinetochore-related complex subunit 3), a protein involved in mitotic regulation, negatively correlates with DUSP2 expression and is related to N stage, peritoneal metastasis, and poor prognosis. Mechanistically, SKA3 negatively regulates the tumour suppressor DUSP2 and activates the ERK1/2 pathway to promote GC in vitro (in the MGC-803 and HGC-27GC cell lines) and in vivo. These results indicate that the SKA3–DUSP2–ERK1/2 axis is involved in GC progression and could be a potential therapeutic target for this cancer [43]. The antitumoural role of DUSP2 has also been confirmed in the context of BLCA. Indeed, Zou and colleagues demonstrated both in vitro and in vivo that the overexpression of DUSP2 suppresses cancer progression through the inhibition of ERK1/2 phosphorylation and M2 macrophages chemotaxis [44].

Collectively, all the above studies point to a protective role of DUSP2 in cancer by limiting ERK1/2 activation and tumour progression, identifying this DUSP as a potential therapeutic target across multiple types of tumours.

### 2.3. DUSP3

DUSP3, also referred to as Human Vaccinia H1-related (VHR) phosphatase, is one of the members of the atypical DUSP group. This constitutively expressed nuclear DUSP is active on JNKs and ERK1/2, and is involved in various cellular functions ranging from cell cycle regulation to immune response [91,92].

The first study implicating DUSP3 in cancer showed that DUSP3 KD in HeLa cells, leads to cell cycle arrest at both the G1-S and G2-M transitions, and induces the initial signs of cellular senescence. These effects were JNK- and ERK1/2-dependent, because cell cycle arrest did not occur upon JNK and ERK1/2 inhibition [93].

However, accumulating evidence points to a possible role of DUSP3 as a tumour suppressor. In NSCLC, it has been reported that DUSP3 expression is epigenetically repressed by the histone H3 lysine 36 demethylase KDM2A (also called FBXL11 and JHDM1A), which is frequently overexpressed in NSCLC tumours and cell lines, and the high level of expression of which correlates with poor prognosis. KDM2A overexpression in NSCLC cells with low KDM2A levels increased cell proliferation and invasiveness (H460 and H2122 cells), while KDM2A KD abrogated tumour growth and invasiveness in NSCLC (H1792 cells) xenografts. These effects were likely supported by ERK1/2 activation following KDM2A-induced DUSP3 repression [45]. Another study showed that DUSP3 may play a role in miR-1915-3p-driven development of BC. Indeed, overexpression of miR-1915-3p, the level of which is increased in BC patients compared with healthy volunteers, determined a reduction of the target gene DUSP3 while increasing the phosphorylation of ERK1/2. Functional in vitro experiments showed that miR-1915-3p enhanced cell proliferation and migration, in keeping with the evidence that patients with infiltrating carcinoma or lymph node metastasis have a higher serum level of miR-1915-3p than patients with in situ carcinoma or without lymph node metastasis [94]. A tumour-suppressive role for DUSP3 has been further supported by a recent study demonstrating that L-methionine epigenetically induces DUSP3 expression, resulting in decreased activation of the ERK1/2 pathway and enhanced sensitivity to sorafeninb in HepG2 cells and a rat model of sorafenib-resistant HCC [46].

Although initially reported as a possible tumour-supporting signal in cervical cancer cells, DUSP3, mainly through the regulation of ERK1/2, emerges as an interesting signalling molecule in other types of cancer, such as BC, NSCLC, and HCC.

### 2.4. DUSP4

DUSP4/MKP-2 is a nuclear DUSP that is responsible for the dephosphorylation and inactivation of ERK1/2, JNK, and p38 and is encoded by a highly inducible gene, which is rapidly upregulated in response to both mitogenic and stress stimuli [53,95]. DUSP4 is one of the most closely related enzymes of the well characterized DUSP1, and its role in cancer progression gained importance in the last decade.

Early evidence of the involvement of DUSP4 in cancer was provided by Yip and colleagues, who showed that PDAC cells expressing oncogenic KRAS upregulates the expression of DUSP4 to counteract ERK1/2 pathway activation [96]. Along this line, a later study demonstrated that DUSP4 has an antitumoural role in the progression to invasive PDAC. Expression profile studies in PDAC cell lines (PANC-1, KP4-1, and MIApaca2) identified DUSP4 as one of the most downregulated genes upon 8p11.22-ter loss. Restoration of DUSP4 expression did not affect the proliferation, but impaired invasiveness in vitro. Xenografts with restored expression of DUSP4 in PANC-1 cells with 8p loss showed longer survival and reduced tumour growth and metastasis compared with mice transplanted with WT PANC-1. The authors also demonstrated that the activation of ERK1/2 following the decrease in DUSP4 is responsible for enhanced resistance to anoikis and the invasiveness of pancreatic carcinomas [48]. A possible oncosuppressor role of DUSP4 has also been identified in head and neck SCC (HNSCC), in which the expression of G9A/EHMT2 histone methyltransferase was found to be associated with tumour growth and poor prognosis. Orthotopic xenografts using FaDu HNSCC cells with inducible KD of G9a showed reduced growth and increased autophagy. In vitro, G9A KD led to increased expression of DUSP4 and to decreased phosphorylation of ERK1/2 as well as to reduced cell growth (FaDu and SAS HNSCC cell lines) [97]. Likewise, DUSP4 showed antitumour effects in GC, where IHC analysis of tumour specimens showed that DUSP4 downregulation is associated with enhanced tumour size, depth of invasion, and distant metastasis. Furthermore, DUSP4 overexpression in GC cell lines (SGC-7901 and HGC-27) reduced cell viability and invasive potential and induced apoptosis and cycle arrest. Interestingly, in the same study, it was shown that sanguinarine, a natural antitumoural benzophenanthridine alkaloid extracted from plants of the Papaveraceae family, inhibits the growth and invasion of GC cells through the DUSP4/ERK1/2 pathway [98]. In BC, low expression of DUSP4 positively correlates with a high Ki-67 score following neoadjuvant chemotherapy and with basal-like BC subtypes. In BC cell lines (BT-549, MDA-MB-231, MDA-MB-436, T47D, and SUM159PT), low DUSP4 expression correlated with increased ERK1/2 phosphorylation. DUSP4 overexpression inhibited ERK1/2 phosphorylation and reduced cell viability, while DUSP4 KD attenuated docetaxel toxicity. Additionally, MEK1/2i (selumetinib, U0126, or CI1040) improved chemotherapy-induced apoptosis [99]. A later study directed to deepen this latter effect showed that low DUSP4 expression is associated with high MEK1/2 phosphorylation in BC specimens and cell lines (MDA-MB-231, ZR751, MDA-MB-468, SUM159PT, BT549, MFM223, and HCC1143) endowed with cancer stem cell characteristics and that DUSP4 KD enhances mammosphere formation [100]. Another study analysing 22 different BC cell lines showed that DUSP4 is downregulated in TNBC cell lines compared to other BC subtypes. Moreover, TNBC cells were found to be more sensitive in vitro to the anti-proliferative effects of simvastatin, a statin used in clinics to reduce hyperlipidaemia [101]. Importantly, simvastatin determined an increase in DUSP4 in TNBC compared with non-TNBC cell lines. Accordingly, DUSP4 overexpression, like simvastatin, reduced cell viability, while DUSP4 KD determined opposite effects. The expression of DUSP4 was accompanied by the reduction of ERK1/2 phosphorylation, while DUSP4 inhibition resulted in increased ERK1/2 phosphorylation, suggesting the involvement of ERK1/2 in the above effects [102]. Along this line, another study reported that DUSP4 KD stimulates ERK1/2 and p38 MAPK pathways, stem-like properties, and metastatic capacity both in vitro in ER-positive MCF-7 cells and in vivo in xenografts of mice injected with DUSP4-KD cells derived from ER-positive and TNBC patients [47]. In another work, it was demonstrated that DUSP4 is upregulated in MCF-7 cells after treatment with tamoxifen and in tamoxifen-resistant MCF-7 cells, along with increased ERK1/2 phosphorylation. However, DUSP4 overexpression did not affect tamoxifen sensitivity of MCF-7 cells but suppressed E2-induced (17-beta-estradiol) proliferation and abrogated ERK1/2 phosphorylation with respect to control cells. These data suggest that ERK1/2 and DUSP4 are involved in tamoxifen sensitivity. The authors speculate that tamoxifen may initially activate ERK1/2, inducing DUSP4 expression to reduce phospho-ERK1/2 levels. However, if phospho-ERK1/2 levels are too high, DUSP4 may be unable to fully dephosphorylate them, potentially contributing to tamoxifen resistance [103]. A recent study showed that overexpression of oncogenic NRAS (G12V) alone is enough to induce hepatocellular carcinoma (HCC) in mice. During tumour progression, the RAS–RAF–MEK1/2–ERK1/2 pathway was consistently activated, along with upregulation of DUSP4, DUSP6, and the cancer stem cell marker CD133. In line with the notion that DUSP4 and DUSP6 act as negative feedback regulators, limiting MAPK signalling to prevent oncogene-induced senescence, their silencing increased cell proliferation and ERK1/2 activation in liver cancer cells. The study also identified a correlation between RAS pathway activation and DUSP4/6 expression in both HCC and cholangiocarcinoma (CCA), suggesting broader relevance. These findings identify DUSP4 and DUSP6 as biomarkers of RAS activity and potential therapeutic targets in RAS-driven liver cancers [104].

At variance with the above studies, a study from Cagnol and colleagues suggests that the negative feedback exerted by DUSP4 on the duration and magnitude of nuclear ERK1/2 activation may support intestinal tumourigenesis. In particular, the expression of either KRAS(G12V) or BRAF(V600E) in normal intestinal epithelial crypt cells (IECs, IEC-6) stimulated ERK1/2 activity and morphological transformation. ERK1/2 phosphorylation was restricted to the cytoplasm, while nuclear ERK1/2 dephosphorylation was found to be tightly correlated with the rapid MEK1/2-dependent expression of DUSP4. Likewise, in human CRC cells (SW480), ERK1/2 phosphorylation was confined within the cytoplasm, while treatment with pervanadate, a tyrosine phosphatase inhibitor effective on DUSP, was necessary to activate ERK1/2 in the nucleus. Accordingly, DUSP4 mRNA was found to be highly expressed, in an MEK-dependent manner, in all CRC cells analysed [105].

On the whole, DUSP4 plays an important role in cancer onset and progression, possibly with the exception of CRC. Thus, DUSP4-modulating agents aimed at increasing its expression may provide new therapeutic options for the treatment of different types of cancer, including PDAC, GC, HNSCC, and BC.

### 2.5. DUSP5

The expression of DUSP5, also referred to as VH1-like phosphatase-3 (VH3), is induced by either heat shock or growth factor stimulation in mammalian cells [106,107,108]. At variance with other inducible DUSPs, DUSP5 is highly selective in its ability to bind and inactivate ERK1 and ERK2 in vitro and in vivo and is localized in the nucleus [109]. One of the first studies linking DUSP5 to cancer reported that its promoter is hypermethylated in GC tissues but not in healthy gastric mucosa. Importantly, GC patients with hypermethylated DUSP5 promoter exhibited significantly shorter survival than those without hypermethylation. Overexpression of DUSP5 in DUSP5-low expressing GC (MKN-74 and SNU-638) cells decreased growth and colony-formation ability and resulted in nuclear ERK1/2 dephosphorylation. This study therefore provided evidence that DUSP5 suppression by promoter hypermethylation results in increased ERK1/2 phosphorylation that supports cell proliferation, possibly driving gastric carcinogenesis. The authors also proposed that DUSP5 promoter methylation may serve as a prognostic marker for GC [110]. Another paper, supporting the antiproliferative role of DUSP5, showed that DUSP5 KO mice show increased sensitivity to HRas (HRasQ61L)-driven papilloma formation in the 7,12-dimethylbenz[a]anthracene/12-O-tetradecanoylphorbol-13-acetate (DMBA/TPA) model of skin carcinogenesis. Indeed, compared with controls, tumours from DUSP5 KO mice showed increased phosphorylation of nuclear ERK1/2 that was associated with the support of tumour growth [49]. Along this line, an IHC study showed that DUSP5 is downregulated in PC with respect to normal tissues and that lower DUSP5 expression is associated with cancer progression and poor prognosis, pointing to the ability of DUSP5 to act as an oncosuppressor in PC [111]. Another IHC study showed that DUSP5 is also downregulated in CRC specimens compared to normal tumour-surrounding tissue. In the same study, it was obtained in vitro evidence that DUSP5 is a negative feedback regulator of the ERK1/2 pathway upon EGF stimulation in the LIM1215 CRC cell line. Interestingly, the overexpression of DUSP5 in mouse intestinal epithelium did not affect ERK1/2 signalling. Thus, the authors suggested that DUSP5 does not regulate ERK1/2 signalling in the normal intestinal epithelium, while showing limited tumour suppressive activity in CRC [112]. In a more recent study, quercetin was shown to increase the expression of DUSP5 following binding of the Serum Response Factor (SRF) transcription factor at the DUSP5 promoter to reduce ERK1/2 activity in the nucleus and cell proliferation, resulting in a tumour-suppressive effect [113]. In another study, DUSP5 emerged as a possible oncosuppressor in glioma by counteracting the protumoural role of the p68 RNA helicase [114], which had been previously demonstrated to be overexpressed in glioma and to be correlated with poor overall survival [115]. Indeed, genetic inhibition of p68 led to increased DUSP5 expression and to decreased ERK1/2 phosphorylation. Moreover, both p68 inhibition and DUSP5 overexpression led to a decrease of ERK1/2 phosphorylation and the proliferation, invasion, and migration of the glioma cell line U87 [109]. DUSP5 has also been proven to reduce the proliferation and invasiveness of TNBC MDA-MB-231 and MDA-MB-468 cell lines [116]. In contrast to all the studies above, a recent work showed that DUSP5 is overexpressed in BRAF-mutant thyroid cancer cells and that DUSP5 KD reduces proliferation and migration. It has also been demonstrated that DUSP5 KD inhibits ERK1/2 phosphorylation and that its KD enhances the antitumour effect of sorafenib [50].

On the whole, almost all the above studies indicate that DUSP5 counteracts cancer onset and development in a number of different types of tumours, so that agents including epigenetic drugs that are able to increase/restore its expression should be sought after.

### 2.6. DUSP6

DUSP6/MKP-3 is an inducible cytosolic DUSP that targets ERK1/2 [117,118,119,120,121,122]. Few lines of evidence link DUSP6 to the modulation of ERK5 signalling in cancer cells. However, whether ERK5 is a substrate of DUSP6 has not been demonstrated. DUSP6 is involved in many physiological functions, including DNA damage repair and stress responses, as well as cancer onset and progression [8,123]. Similar to other inducible DUSPs, ERK1/2 activation supports both transcriptional and post-transcriptional modifications that collectively increase DUSP6 protein levels, resulting in a negative feedback in both normal and neoplastic cells that avoids excessive activation of ERK-dependent signals, including cell proliferation [124,125,126]. Conversely, DUSP6 may also function as a tumour-supporting protein by helping tumour cells to adapt to an abnormally enhanced ERK signalling [127].

A number of studies have demonstrated a clear role of DUSP6 as an oncosuppressor in different types of cancer, lung cancer being among the most studied. In silico analysis using Gene Expression Omnibus (GEO) and The Cancer Genome Atlas (TCGA) databases revealed an association between a low expression of DUSP6 and a poor prognosis in NSCLC patients. In agreement with these clinical data, DUSP6 emerged as a key player in cell migration and tumour growth, as well as in the downregulation of several genes involved in epithelial-to-mesenchymal transition (EMT). Referring to the latter, RNA-seq identified EGFR, TGF-β, and WNT signalling pathways among the genes upregulated in DUSP6 KD cells. In these cells, activation of the EGFR signalling was proven to occur via ERK5 and not via ERK1/2. However, whether this effect was due to a direct dephosphorylation of ERK5 by DUSP6 was not investigated [61]. Interestingly, a later IHC study performed using human specimens from 66 human lung adenocarcinoma (LUAD) showed that DUSP6 expression positively correlates with a lower expression of the proliferation marker KI67, and lower histological grade, likely acting as a negative feedback regulator of ERK1/2 in LUAD progression [128]. In support of this hypothesis, a later study reported that DUSP6 is overexpressed in human LUAD cell lines and impairs ERK1/2 activation [129]. Notably, aberrant ERK1/2 activation is a frequent event in NSCLC as a consequence of constitutive activating mutations of either EGFR or KRAS [130]. On the other hand, synthetic lethality has been described when mutant KRAS and EGFR proteins are co-expressed in LUAD cells (PC9, H358 and H1975), revealing the biological basis for the observed mutual exclusivity of KRAS and EGFR mutations [131]. Interestingly, DUSP6 might be involved in the toxicity elicited as above. By combining pharmacological and genetic approaches to inhibit KRAS or EGFR, it was shown that downregulation of DUSP6 (with siRNA or BCI) or overexpression of ERK1/2 can lead to RAS-induced toxicity in LUAD cell lines. This study provided evidence that tumours harbouring mutant RAS limits ERK1/2 activity to prevent excessive toxicity, despite supporting tumour growth. It also identified a protumoural role of DUSP6 in LUAD cancer, showing that DUSP6 may prevent synthetic lethality arising from the co-expression of mutant KRAS and EGFR proteins [129]. In another work, it has been reported that DUSP6 expression tracks in tandem with low ERK1/2 phosphorylation in both NSCLC patient-derived samples and cell lines. Mechanistically, DUSP6 KD in NSCLC cells expressing high levels of DUSP6 (H441 cells) significantly increased ERK1/2 phosphorylation and cell proliferation, whereas overexpression of DUSP6 in low DUSP6-expressing cells (H1975) significantly reduced ERK1/2 phosphorylation and cellular proliferation and promoted apoptosis. These results indicate that DUSP6 expression is regulated by ERK1/2 signalling and that DUSP6 exerts antitumour effects via negative feedback regulation of ERK1/2 in NSCLC [132]. In another study performed in LUAD cells, the repression of DUSP6 induced the overactivation (i.e., phosphorylation) of Translation Initiation Factor 2 (eIF2) and ERK1/2. Using xenografts of LUAD cells (KRAS G12D eIF2αS/S and eIF2αA/A), the authors established a connection between ERK1/2 phosphorylation and the increased phosphorylation of eIF2. In particular, they demonstrated that the repression of DUSP6 is caused by activation of eIF2, which in turn leads to the activation of ERK1/2. Consistently, either genetic or pharmacological inhibition of eIF2 determined an upregulation of DUSP6 that impairs the formation of LUAD both in vitro and in vivo [133]. In line with the above observed tumour suppressor roles of DUSP6, in A549 and H1975 NSCLC cells, inhibition of GPx3, a ROS scavenger, determined ROS-induced inactivation of DUSP6 as well as an increase in ERK1/2 phosphorylation and cell proliferation [134]. Regarding the possible mechanisms involved in ERK1/2-mediated DUSP6 expression in NSCLC, it has been reported that ETS1, a well-known nuclear target of activated ERK1/2, positively modulates DUSP6 expression by binding to its promoter region [132]. Furthermore, a recent study reported that the transcription factor ZNF251 inhibits the expression of DUSP6 by directly binding to its promoter region and activates ERK1/2 signalling in NSCLC cell lines (A549, H520, H23, H460, and SPC-A-1) and in a mouse model with a KRAS mutation (G12D) [135].

As for melanoma, DUSP6 overexpression in human melanoma cells (A375) reduced tumour xenograft growth in mice. In the same study, DUSP6 levels were not found to be correlated with a better survival of patients with thick melanomas. Furthermore, DUSP6 overexpression in immortalized murine melanocytes enhanced anchorage-independent growth and invasive ability, pointing to a protumoural role of DUSP6, at least in normal murine cells [136]. However, a recent work performed using the B16-F10 melanoma cell line demonstrated that hinokitiol, a plant-derived metal chelator, induces an increase in DUSP6 along with a DUSP6-dependent decrease of ERK1/2 phosphorylation and of cell proliferation [137]. In another study, the same authors identified a relevant role of DUSP6 in cisplatin resistance. Indeed, cisplatin increased the expression of the DNA repair protein ERCC1 and XPF, along with a reduction in DUSP6 protein. These effects were dependent on ERK1/2 because a MEK1/2 inhibitor (PD0325901) prevented their occurrence. DUSP6 overexpression prevented cisplatin-dependent induction of both ERCC1 and XPF and restored sensitivity to cisplatin, confirming that DUSP6 has an antitumoural role in melanoma [138]. In a later work based on IHC of melanoma specimens, it was found that DUSP6 protein, as well as STAG2 and STAG3, two cohesin complex components, are decreased following treatment with BRAF or MEK (dabrafenib, trametinib, andvemurafenib) inhibitors. Consistently, melanoma cell lines (A375, Mel1617, WM902, WM983, and M14) resistant to BRAFi or MEKi showed reduced expression of STAG2 and STAG3, and their KD led to a further decrease of DUSP6 as well as of BRAFi sensitivity, along with an increase in ERK1/2 phosphorylation. The same effects were observed using A375 and WM902 xenografts in mice, further confirming the protective role of DUSP6 in melanoma [55]. Finally, overexpression of DUSP6 was shown to counteract the protumoural role of Mir-211 in melanoma. Indeed, A375 cells overexpressing Mir-211 showed enhanced ability to form xenografts into SCID mice. Mir-211 overexpression led also to an increase of ERK5 phosphorylation and to the downregulation of DUSP6 [139]. Overexpression of DUSP6 in A375/211 cells led to the reduction of tumour growth of xenografts in mice. In the same in vivo model, they also demonstrated that Mir-211 overexpression confers resistance to vemurafenib (BRAFi) and cobimetinib (MEKi) through the activation of ERK5, a MAPK supporting melanoma growth [139], suggesting that DUSP6 may potentially prevent this undesirable effect [52].

Loss of DUSP6 expression, at either the mRNA or protein level, has been proven to correlate with high ERK1/2 phosphorylation in primary human OC cells (OV2008, C13*, A2780s, A2780cp, DOV13-5, OVCAR3, SK/OV3, OV420, OV429, and OV433). Loss of DUSP6 protein was proteasome dependent, as a consequence of high intracellular ROS. DUSP6 KD resulted in increased ERK1/2 activity, cell proliferation, anchorage-independent growth, and resistance to cisplatin. Conversely, overexpression of DUSP6 in DUSP6-deficient OC cells significantly reduced ERK1/2 activity and inhibited cell proliferation, anchorage-independent growth, and tumour development in nude mice. Furthermore, DUSP6 overexpression sensitized OC cells in vitro and xenografts in mice (A2780 cell line) to cisplatin-induced apoptosis. On the whole, accumulation of ROS during OC progression may cause the degradation of DUSP6, which in turn leads to aberrant ERK1/2 activation, thus contributing to the tumourigenicity and chemoresistance of human OC cells [51]. In support of a relevant role of ubiquitination in DUSP6 downregulation in OC cells, it has been reported that gene amplification of the oncoprotein tripartite motif-containing 59 (TRIM59), an E3 ubiquitin ligase, is associated with decreased expression of DUSP6 and a shorter overall survival in OC. Genetic inhibition of TRIM59 in different OC cell lines (SK/OV3 and OVCAR3) led to a decrease in ERK1/2 phosphorylation and to an increase in DUSP6 protein but not mRNA levels [140]. Along this line, a study dealing with pancreatic cancer investigated the effects of the loss of the DUSP6-dependent negative feedback on ERK1/2. By IHC analysis, the authors reported downregulation of DUSP6 in invasive carcinoma. Overexpression of DUSP6 in invasive pancreatic cancer cells (PCI-35 and PK-8) resulted in a reduction in phosphorylated ERK1/2 and resulted in cell growth suppression and apoptosis [141]. An oncosuppressive role for DUSP6 has also been described in CRC. Depletion of DUSP6 in colonic epithelium in mice caused an increase in the levels of ERK1/2 phosphorylation and KI67. It also induced colonoid (i.e., colon-derived organoid) development and intestinal tumourigenesis in Apc Min/+ mice. Additionally, genetic silencing of DUSP6 in CRC cell lines (HT29 and HCT116) determined an increase in ERK1/2 phosphorylation as well as cell migration and invasion ability in vitro [53]. In line with the above work, another recent study demonstrated that the interaction of the protein gasdermin B (GSDMB) with insulin-like growth factor 2 mRNA-binding protein 1 (IGF2BP1) leads to an increase in DUSP6 protein levels and subsequent inhibition of ERK1/2 phosphorylation. In both cell lines (HT-29) and mice, it emerged that the increased expression of GSDMB and subsequently that of DUSP6 reduced cell proliferation and tumour growth [57]. In non-solid tumours, Xu and colleagues recently showed that in acute myeloid leukaemia (AML), the Wilms tumour 1 (WT1) protein can activate DUSP6 transcription, but only in the presence of the RUNX1::RUNX1T1 fusion (also known as t(8;21)). This leads to inhibition of the ERK1/2 pathway. In contrast, when the fusion is absent, WT1 fails to induce DUSP6 expression, resulting in increased ERK1/2 phosphorylation and promoting leukemogenesis [58]. The role of DUSP6 in tumourigenesis has also been investigated in ESCC and nasopharyngeal carcinoma (NPC). Significant loss of DUSP6 was observed in 100% and 71% of ESCC and NPC cell lines, respectively. DUSP6 expression was downregulated in 40% and 75% of tumour specimens of ESCC and NPC, respectively, compared to normal counterparts. Notably, tissue microarray analysis revealed a clinical association of DUSP6 expression with better patient survival. Overexpression of DUSP6 in ESCC (SLMT-1, KYSE70, and KYSE450) and NPC cell lines (HONE1, HNE1, CNE2, and SUNE1) reduced tumour growth and invasiveness in vitro and in xenografts in mice. This study thus provides evidence of the functional impact of DUSP6 in tumourigenesis and metastasis of ESCC and NPC [60]. Along this line, another study performed in endometrial cancer cells, showed that DUSP6 overexpression in the Ishikawa cell line led to a decrease in ERK1/2 phosphorylation and E-cadherin expression. Additionally, DUSP6 inhibited cell growth, invasion and migration abilities [142]. Despite this study providing evidence that DUSP6 may counteract endometrial cancer progression, it has not been reported whether DUSP6 expression is reduced or increased in this type of tumour. However, hypermethylation of the DUSP6 promoter, which may determine a reduction of DUSP6 expression, has been reported to be a rare event in endometrial cancer [143]. Furthermore, another recent work supported the tumour suppressor role of DUSP6 in cervical cancer, demonstrating that the protein ribosomal L22-like 1 (RPL22L1) is overexpressed in this type of tumour and is capable of binding and sequestering DUSP6. This led to increased phosphorylation of ERK1/2 and conferred resistance to sorafenib as demonstrated with both in vitro and in vivo assays [56]. DUSP6 seems to have an oncosuppressor role also in renal cell carcinoma (RCC) as it was demonstrated by Liu and colleagues. They showed that DUSP6 downregulation correlates with advanced disease stage and poor clinical outcome. They also demonstrated that hepatocyte nuclear factor 4 alpha (HNF-4α) regulates DUSP6 expression and that the treatment with calcium saccharate (CAS) can upregulates DUSP6 expression. Moreover, CAS treatment sensitized both parental and sunitinib resistant RCC cells (786-O) to sunitinib both in vitro and in vivo [59].

Several lines of evidence indicate a protumoural role for DUSP6 in other types of cancer. DUSP6 is overexpressed and is linked to a poorer overall survival in GC. In GC cell lines (BGC823, SGC7901, and cisplatin-resistant SGC7901), DUSP6 KD inhibited proliferation, migration, and invasion and induced apoptosis. Consistently, the DUSP inhibitor BCI reduced invasion, migration, and proliferation and enhanced cisplatin cytotoxicity in GC cells in vitro. Despite increasing ERK1/2 phosphorylation, BCI treatment led to the reduction in mRNA levels of some ERK1/2-regulated genes. More importantly, BCI enhanced the antitumour effects of cisplatin in GC cell line-based and patient-derived xenograft models [54]. Another study performed in HCC showed that DUSP6 expression and ERK1/2 phosphorylation were higher in tumour samples compared to peritumoural and normal tissue, as determined by IHC. Additionally, higher expression of DUSP6 in tumour tissue correlated with HCC recurrence after resection [144]. Another retrospective IHC study showed that the expression of ERK1/2, DUSP6, c-Fos, c-Myc, cyclin D1, and PCNA is increased in papillary thyroid carcinoma (PTC) compared to benign neoplasms. Moreover, high DUSP6 expression was associated with the level of ERK1/2 expression and with high-risk biological features, including tumour size. Consistent with the protumoural role of DUSP6 in PTC, DUSP6 silencing decreased cell viability and migration of anaplastic thyroid carcinoma FRO cells [145]. In another work performed in PTC, expression of RET/PTC3, HRASV12, and BRAFV600E in PCCL3 cells determined an increase in DUSP5 and DUSP6 mRNA as well as MEK1/2 and ERK1/2 phosphorylation. Inhibition of MEK, but not of PI3K/AKT, partially prevented the above increase in DUSP5 and DUSP6. Using two BRAFV600E thyroid carcinoma cell lines (BCPAP and 8505c), the same authors showed that genetic inhibition of DUSP5 and DUSP6 did not affect cell proliferation or ERK1/2 phosphorylation, while reducing cell migration and invasion [146]. Upregulation of DUSP6 has been reported to show a tumour-promoting role in human glioblastomas. Indeed, DUSP6 mRNA and protein levels are upregulated in primary and long-term cultures of human glioblastoma cells (U251, T98G, and U87MG) compared to normal human astrocytes. DUSP6 overexpression led to reduced ERK1/2 phosphorylation as well as to an increase in the cisplatin resistance of U87MG cells in vitro and in vivo and was associated with increased xenografts growth [147]. Finally, a study conducted on sarcoma cells (NCC_CDS_X1) revealed that the oncoprotein CIC-DUX binds to DUSP6, causing a decrease in ERK1/2 phosphorylation and consequent overexpression of the oncoprotein. Pharmacological (BCI) or genetic (shRNA or siRNA) inhibition of DUSP6 leads to an increase in ERK1/2 activation and a decrease in the level of CIC-DUX, resulting in increased apoptosis. This may represent an innovative therapeutic approach to targeting CIC-fused sarcoma [148].

As far as BC is concerned, the role of DUSP6 has been more controversial. Gene expression profile analysis provided evidence that high DUSP6 mRNA levels are associated with resistance to the anti-oestrogen tamoxifen in both patient samples and cell lines. In line with this fact, overexpression of DUSP6 rendered ERα-positive BC cells (MCF7) resistant to tamoxifen. DUSP6 overexpression was also associated with lower levels of ERK1/2 phosphorylation. The MEK inhibitor PD98059 blocked the proliferation of tamoxifen-resistant cells, pointing to a role of ERK1/2 activation in the above process. These results suggested that tamoxifen increases ERK1/2 activity via the loss of DUSP6 and that this activity remains sensitive to MEK inhibition. Therefore, patients with tamoxifen-resistant disease and elevated DUSP6 may be markedly sensitive to MEK inhibitors [149]. A possible protumoural role of DUSP6 has also been reported in TNCB. In particular, DUSP6 expression was higher in the MDA-MB-231 TNBC cell line than in patient-derived ER-positive BC cells. In the former, DUSP6 KD (siRNA) reduced cell growth, migration and invasion, and the same result was obtained using mir-145 to inhibit DUSP6. Whether DUSP6 expression was correlated to ERK1/2 phosphorylation was not addressed [150]. The above results are at variance with those of the work carried out by Bergholz and colleagues with MDA-MB-231 cells, where DUSP6 emerged as an inhibitor of tumour invasiveness. Indeed, the authors showed that overexpression of the p63 isoform ΔNp63α inhibits cell invasion by eradication of ERK1/2 signalling via DUSP6 in vitro and in vivo [151]. Importantly, the BCI derivative BCI-215 has been reported to induce apoptosis and to sensitize MDA-MB-231 cells to immune cell killing [30]. Another study identified the increased expression of DUSP6 as a key factor in the progression of secondary acute myeloid leukaemia (sAML) by promoting JAK-STAT and ERK signalling, inflammatory cytokine production, and resistance to JAK2 inhibitors. Targeting DUSP6 inhibited these pathways, reduced disease severity, and suppressed sAML development in preclinical models, including Jak2V617F and MPLW515L mouse models, without affecting healthy cells, also indicating a protumoural role of DUSP6 in non-solid tumours [62].

DUSP6 is also implicated in chemoresistance in several cancers. In OC, elevated DUSP6 expression correlates with cisplatin resistance by attenuating ERK1/2 signalling as observed in patient tumour samples and in cancer cell lines upon overexpression [152]. At variance with this study, other authors showed that DUSP6 KD resulted in increased ERK1/2 activity, cell proliferation rate, anchorage-independent growth ability, and resistance to cisplatin in the same cancer model [131]. In an article by Hrustanovic et al., the authors reported that DUSP6 KD promoted resistance to ALK inhibitors in vitro as well as in individuals with EML4- and ALK-positive lung adenocarcinoma by reactivation of ERK1/2 signalling [153]. This underscored the dual and tissue-specific functions of DUSP6, whose contribution to chemoresistance may vary across different tumour types. Nevertheless, DUSP6 inhibition may be exploited as a potential strategy to overcome chemoresistance. Accordingly, the compound BCI has shown promise in re-sensitizing resistant cells to chemotherapy in gastric cancer and ovarian cancer [142,154]. Moreover, in HER2-positive BC, DUSP6 inhibition with BCI, its derivative BCI-215, and FTY-720 can overcome resistance to HER2 inhibitors by targeting the HER3 signalling pathway, which is often involved in the development of resistance [155].

On the whole, DUSP6, one of the most studied DUSPs in cancer, has been shown to have both protumoural and antitumoural properties depending on the type of cancer analysed. In particular, it exerts antitumoural effects in OC, melanoma, and NSCLC, while showing a protumoural role in GC, sarcoma, and glioblastoma. Furthermore, it emerged that DUSP6 could be used as a marker for the detection of some tumours, and it can also be used as a target for the treatment of others. Of note, it is important to continue the study of this DUSP and its signalling to better understand its role in other types of cancer, especially in those tumours, like BC, where it has been shown to have a controversial behaviour.

### 2.7. DUSP7

DUSP7/MKP-X, also known as PYST2, is a cytoplasmic phosphatase that inactivates ERK1/2 [110,156,157,158]. Like other DUSPs, DUSP7 binds to its targets through a conserved arginine-rich kinase interaction motif, which is located amino-terminally of the phosphatase domain [159]. Although there are few reports linking DUSP7 to cancer, current evidence suggests that it may act predominantly as a tumour suppressor. In the MCF-7 BC cell line, oestrogen deprivation induces the expression of the long non-coding RNA LincRNA regulator of reprogramming (Linc-RoR) and activates ERK1/2. This activation is Linc-RoR-dependent because it is abolished upon Linc-RoR KD. Importantly, Linc-RoR KD caused an increase in DUSP7 protein levels as well as a decrease in ERK1/2 phosphorylation and cell proliferation. In keeping with a possible protective role of DUSP7 in ER-positive BC, in silico data indicated that reduced DUSP7 expression correlates with a worse prognosis in ER-positive BC patients and with a shorter relapse-free survival [160]. Another study performed using GC cells addressed the hypothesis that asporin (ASPN), a member of the small leucine-rich repeat proteoglycan (SLRP) family of proteins [161], regulates the proliferation of GC cells through the downstream target proteasome 26S subunit non-ATPase 2 (PSMD2). PSMD2 participates in the downregulation of DUSP7, WIP1, and PTEN. For this reason, genetic inhibition of ASPN yielded an increase in DUSP7, WIP1, and PTEN and the inactivation of the MAPK signalling pathway along with a reduction in cell proliferation [162]. In line with an antitumoural action of DUSP7, a recent work demonstrated that in CRC with SMAD4 deletion, SETD2, a histone methyltransferase, promotes the transcription of DUSP7, leading to inactivation of the RAS/ERK pathway and reduced tumour growth [63]. In contrast with the above studies, a recent work performed in CRC identified the microRNA let-7c-5p as a tumour suppressor in HCT8 cells. Its overexpression reduced the levels of DUSP7 and also the phosphorylation of ERK1/2, leading to the increased expression of the pro-apoptotic marker BAX, shedding light for the first time on a protumoural role of DUSP7 [163].

Thus, DUSP7 seems to exert an antitumoural role in both BC and GC malignancies, while its role in colorectal cancer appears to be controversial. Nevertheless, considering the paucity of studies focused on the role of DUSP7 in cancer, further studies are needed to further characterize its role in cancer.

### 2.8. DUSP9

DUSP9/MKP-4 is a cytosolic DUSP that inactivates ERK1/2 but also JNK and p38, and belongs to a subfamily of three closely related cytoplasmic dual-specificity MAPK phosphatases, which includes the ERK1/2-specific enzymes DUSP6 and DUSP7 [164]. As for its possible antitumoural effect, in a clonal model of epidermal carcinogenesis, DUSP9 was found to be downregulated at initiation and lost at malignant conversion. Reconstitution of DUSP9 expression in H1299 SCC cells led to cell death and tumour growth suppression, as a consequence of G(2)-M associated cell death and microtubule disruption [165]. In line with the antitumoural role of DUSP9 in this cancer, another study reported that DUSP9 was significantly downregulated in CRC tissues compared with peritumoural ones. Additionally, transcriptomic profiling performed in CRC SW480 cells revealed that DUSP9 KD was associated with activation of ERK1/2 signalling [166]. Other studies reported that DUSP9 KD may be associated with reduced cell proliferation. In particular, it was found that the expression of DUSP9 is upregulated upon retinoic acid receptor (RAR) activation. Indeed, RAR, which promotes DUSP9 expression through direct binding to its promoter region, increases spontaneous differentiation of CRC cells, while ERK1/2 activation suppresses it, favouring proliferation. DUSP9 KD significantly delayed the differentiation of CACO-2 cell line, pointing to a role of RAR/DUSP9 signalling in supporting CRC differentiation. Thus, the authors provided evidence that RAS/ERK1/2 and RAR signalling pathways antagonistically interact with each other to regulate CRC cell fate [167].

A potential protumourigenic role of DUSP9 has been suggested by findings showing that HIF1-dependent regulation of MAPK signalling via DUSP9 contributes to the enrichment of chemotherapy-induced BC stem cells (BCSC) in MDA-MB-231 and SUM-159 TNBC cells. Specifically, chemotherapy was shown to upregulate DUSP9 and downregulate DUSP16 in a HIF-1-dependent manner, leading to ERK1/2 inhibition and p38 pathway activation. ERK1/2 inhibition promoted the transcriptional induction of the pluripotency factor Nanog, while p38 activation stabilized Nanog and Klf4 mRNA. Together, these events supported the maintenance of the BCSC phenotype. Notably, inhibition of HIF-1 or p38 signalling blocked chemotherapy-induced expression of pluripotency factors and prevented BCSC enrichment [168].

The above literature is limited and quite discordant, providing evidence of both protumoural and antitumoural roles regarding DUSP9, even in the same type of cancer. For this reason, DUSP9 is considered an ambiguous phosphatase in the context of cancer onset and development. Thus, further studies are needed to dissect, possibly in a context-specific manner, its possible exploitation as a biomarker and/or a target for cancer therapy.

### 2.9. DUSP10

DUSP10/MKP-5, located in both cytoplasm and nucleus, selectively dephosphorylates p38 and JNK, showing minor activity towards ERK1/2 [169]. It is involved in the regulation of cell proliferation, differentiation, and migration [42,170]. Interestingly, DUSP10 has been proven to interact with and to retain ERK1/2 in the cytoplasm, thus preventing ERK1/2-dependent transcription. These findings reveal a novel function of DUSP10 as a scaffold protein within the ERK1/2 pathway. In the same study, DUSP10 mRNA was found to be frequently upregulated in CRC, but not in lung carcinoma, suggesting a potential association with the malignant phenotype of CRC [171]. With regard to the possible antitumoural role of DUSP10, decreased DUSP10 protein levels along with increased ERK1/2 phosphorylation has been reported in HCC metastasis. KD of the stress-induced tumour suppressor gene TP53INP1, which is downregulated in HCC, led to an increase in the invasion of immortalized normal liver (MIHA) and HCC (MHCC97L) cell lines and metastasis in nude mice injected with MHCC97L-siTP53INP1 cells compared with control MHCC97L cells. Overexpression of DUSP10 in MHCC97L-siTP53INP1 led to ERK1/2 inactivation and a decreased capability of invasion in vitro and metastasis in vivo. Finally, the authors provided evidence that p73 is involved in TP53INP1-dependent regulation of DUSP10 [64].

The limited number of available studies regarding the role of DUSP10 in the modulation of ERK1/2 is in line with the marginal role of DUSP10 in the regulation of these ERKs. DUSP10 inactivated ERK1/2 and showed antitumour properties in HCC, while having opposite effects in CC.

## 3. Conclusions

Evidence about the correlation of altered expression of DUSPs with cancer onset and progression has accumulated over the years, pointing to the possibility to exploit their targeting as an intriguing strategy for cancer treatment. Among the MAPKs that are DUSP substrates, ERK1/2 in particular are implicated in important biological events, including cell proliferation, survival, and migration, and are often deregulated in cancer. Thus, they are frequent targets for molecularly-tailored therapies [172]. Furthermore, although at present there is no evidence that ERK5 is a direct substrate of DUSP1 and DUSP6, both DUSPs have been shown to modulate ERK5 signalling [173,174], pointing to an additional interesting field of research to identify a new possible strategy for cancer treatment. In fact, ERK5 pathway activation is involved in almost all the hallmarks of cancer [121]. In addition, activation of this MAPK is often responsible for the development of resistance to anti-cancer treatments [175]. DUSPs may thus represent an additional target to modulate the activation of ERK5 as a resistance mechanism. Likewise, it is well established that resistance to chemotherapeutic agents often arises from reactivation of MAPK signalling following continuous exposure to the administered drug. Furthermore, as highlighted in this study, such reactivation frequently coincides with deregulation of DUSPs, depending on the tumour type. Therefore, it may be particularly valuable to consider tumour-specific combination therapies in which, alongside MAPK inhibition, simultaneous target the DUSP involved in the regulation of the specific MAPK. This could be achieved either through dual inhibition when the DUSP is overexpressed or through induction of DUSP expression [62,176,177,178].

A critical aspect of targeting DUSPs relies on the fact that these phosphatases can act as either tumour promoters or suppressors according to the cancer type and to the stage of disease. In this regard, computational modelling may provide a useful tool to predict whether inhibition or activation of a specific DUSP impacts cancer aggressiveness [179]. On the other hand, regulation of DUSP activity and protein stability is very complex, providing additional strategies to modulate their activity and expression levels [7]. Furthermore, proteolysis-targeting chimera (PROTAC) could provide additional modulating strategies for DUSPs by either directing specific DUSP proteolysis or by degrading signalling proteins involved in DUSP regulation to boost their expression and/or activation [180].

As for DUSP inhibition, DUSP small molecule inhibitors have been proven to be a promising approach in the contexts in which DUSPs support cancer growth and progression (Figure 2). However, due to the high number of different DUSPs with a relatively well conserved catalytic mechanism, the possibility to obtain highly specific inhibitors seems to be hampered, at least at the moment. Among the DUSPs inhibitors that have demonstrated a good antitumour efficacy, BCI, a benzoil-derivative small-molecule inhibitor of DUSP1 and DUSP6, has been reported to reduce BC, MPNST, LUAD, GC and sarcoma growth [28,29,30]. Noteworthy, the cytotoxic effect of BCI seems to be only in part due to DUSP1/6 targeting [181]. Moreover, NSC 95397, a quinone-based small molecule inhibitor of DUSP1 and DUSP6, reverted the protumoural role of DUSP1 in CC [35,36]. Another interesting approach to exploit the antitumoural effect of DUSPs is the one described by Cheng et al. that administered baculovirus-derived DUSP1 in the medium of Hela cells, obtaining a reduction in their proliferation [85].

Concerning the possibility to boost the antitumoural effect of DUSPs by increasing their expression and/or activation, sanguinarine, a natural antitumoural benzophenanthridine alkaloid, has been reported to upregulate the expression of DUSP4, thus counteracting GC progression [98]. On the other hand, Lee and colleagues have reported that the increased expression of Mir-211 impairs the antitumoural role of DUSP6 in melanoma [52], pointing to the use of non-coding RNAs as an additional strategy to modulate the activity of DUSPs. Another interesting strategy for the modulation of DUSPs is related to the possibility of using small molecules to inhibit or enhance the expression of different transcription factors. Along this line, it has been reported that the transcription factor ETS1 positively modulates DUSP6 expression by binding to its promoter region, leading to a decrease of NSCLC progression [132]. Another study demonstrated that the transcription factor ZNF251 inhibits the expression of DUSP6, activating ERK1/2 signalling and promoting NSCLC growth [135]. On the whole, the available data established the basis for the use of DUSPs as both tumour markers and therapeutic targets in many types of cancer. Despite the fact that DUSP1 and DUSP6 have been studied in detail and there are inhibitors available, further extensive work is needed to better understand the role of all ERK-targeting DUSPs within the various types of tumours and to develop DUSP modulators, both inhibitors and activators, for their utilization in the clinics. Until now, most studies have focused on the role of typical DUSPs in tumour development. However, DUSP3, an atypical DUSP, has been shown to exert antitumoural effects in the majority of published studies. This observation could provide a valuable foundation for future research aimed at exploring the possible contribution of other atypical DUSPs in cancer onset and progression, as well as in the sensitivity and resistance to therapies, in the different types of cancer.

## Figures and Tables

**Table 1:** In vivo evidence of the role of ERK-targeting DUSPs in cancer.

Gene/MKP	Tumour Type (Xenografted Cell Line/Animal Model)	Treatment/Genetic Manipulation (Observed Secondary Effect on DUSPs)	Biological Outcome	Effect on ERKs	Ref
***DUSP1***	CRC (HCT116 and SW480)	DUSP1 overexpression	Increased tumour growth	Reduced pERK1/2	[37]
*DUSP1 and DUSP6*	MPNSTs (S462.TY PDX and xenografts)	DUSP1 and/or DUSP6 KD BCI	Reduced tumour growth	Increased pERK1/2	[38]
*DUSP1*	BLCA (HT1197 and RT112)	AKTi (Increased DUSP1)	Reduced tumour growth	Reduced pERK1/2	[39]
*DUSP1*	HCC	Cx32 KO (Reduced DUSP1)	Increased ethanol-induced HCC	Increased pERK1/2	[40]
*DUSP1*	ESCC (TE-1)	ARNTL overexpression (Increased DUSP1)	Reduced tumour growth	Reduced pERK1/2	[41]
***DUSP2***	PDAC (PANC-1, SW1990, BxPC-3, CFPAC-1)	Mir-361-3p overexpression (Reduced DUSP2)	Increased metastasis	Increased pERK1/2	[42]
*DUSP2*	GC (MGC-803, HGC-27)	SKA3 KD (Increased DUSP2)	Reduced tumour growth	Reduced pERK1/2	[43]
*DUSP2*	BLCA	DUSP2 overexpression	Reduced tumour growth	Reduced pERK1/2	[44]
***DUSP3***	NSCLC (H1792)	KDM2A KD (Increased DUSP3)	Reduced tumour growth	Reduced pERK1/2	[45]
*DUSP3*	HCC (diethylnitrosamine)	DUSP3 overexpression	Reduced tumour growth/Increased sorafenib sensitivity	Reduced pERK1/2	[46]
***DUSP4***	BC (MCF7)	DUSP4 KD	Increased tumour growth	Increased pERK1/2	[47]
*DUSP4*	PDAC (PANC-1)	DUP4 overexpression	Reduced tumour growth	Reduced pERK1/2	[48]
***DUSP5***	Skin cancer (HRasQ61L mice)	DUSP5 KO	Increased papilloma formation	Increased pERK1/2	[49]
*DUSP5*	Thyroid cancer (K1)	DUSP5 KO	Reduced tumour growth	Reduced pERK1/2	[50]
***DUSP6***	Ovarian cancer (A2780)	DUSP6 overexpression	Reduced tumour growth/increased cisplatin sensitivity	Reduced pERK1/2	[51]
*DUSP6*	Melanoma (A375)	DUSP6 overexpression	Reduced tumour growth	Reduced pERK5	[52]
*DUSP6*	CRC *(Apc* Min/+ mice)	DUSP6 KO	Increased intestinal tumourigenesis	Increased pERK1/2	[53]
*DUSP6*	GC (BGC823, SGC7901, SGC7901/DDP on PDX and xenografts)	DUSP6 KO BCI	Reduced tumour growth	Increased pERK1/2	[54]
*DUSP6*	Melanoma (A375)	STAG2 or STAG3 KD (Reduced DUSP6)	Reduced BRAFi sensitivity	Increased pERK1/2	[55]
*DUSP6*	Cervical cancer (HeLa)	RPL22L1 KD (Increased DUSP6)	Reduced tumour growth	Reduced pERK1/2	[56]
*DUSP6*	CRC (GSDMB transgenic mice)	GSDMB overexpression (Increased DUSP6)	Reduced tumour formation	Reduced pERK1/2	[57]
*DUSP6*	AML (Kasumi-1 and HL60)	WT1 overexpression/KD (Increased/reduced DUSP6)	Reduced/increased tumour growth	Reduced/increased pERK1/2	[58]
*DUSP6*	Renal Cell Carcinoma (786-O)	Sunitinib and calcium saccharate (Increased DUSP6)	Reduced tumour growth	Reduced pERK1/2	[59]
*DUSP6*	ESCC (SLMT-1) NPC (HONE1)	DUSP6 overexpression	Reduced tumour growth	Reduced pERK1/2	[60]
*DUSP6*	NSCLC (H460)	DUSP6 KD	Increased tumour growth	Increased pERK5	[61]
*DUSP6*	AML (CD45.1+ C57BL/6J mice)	BCI	Reduced tumour progression	Reduced pERK1/2	[62]
***DUSP7***	CRC	SETD2/SMAD4 KO (Reduced DUSP7)	Increased tumour growth	Reduced pERK1/2	[63]
***DUSP10***	HCC (MHCC97L)	TP53INP1 KD (Reduced DUSP10)	Increased metastasis	Increased pERK1/2	[64]

PDX, patient derived xenograft; pERK1/2, phosphorylated ERK1/2; KO, knock out; KD, knock down; GC, gastric cancer; HCC, hepatocellular carcinoma; MPNST, malignant peripheral nerve sheath tumour; AML, acute myeloid leukaemia; NSCLC, non-small cell lung cancer; PDAC, pancreatic ductal adenocarcinoma; ESCC, oesophageal squamous cell carcinoma; NPC, nasopharyngeal carcinoma; CRC, colorectal cancer; BC, breast cancer; BLCA, urothelial bladder carcinoma. Noteworthy, it cannot be excluded that the biological outcomes are partially due to dephosphorylation of other MAPKs under investigation in the different studies other than ERKs.
